# Parliament and Research

**Published:** 1913-05-24

**Authors:** 


### Parliament and Research.

### THE LOCAL GOVERNMENT BOARD'S GRANTS.

The President of the Local Government Board has authorised the following special researches to be paid for out of the annual Parliamentary grant in aid of investigations into the causes and processes of disease:—

1. A continuation of the Board's inquiry into the causes of premature arterial degeneration, by Dr. F. W. Andrewes.
2. A continuation of the Board's inquiries on insects in relation to disease; Professor Nuttall, F.R.S., on the life cycle of the body louse and bug; Dr. Bernstein and Mr. Hesse on the *empusa muscæ* in flies.
3. A continuation of the Board's inquiry into infantile diarrhœa, by Mr. F. W. Twort and Dr. Edward Mellanby.
4. A continuation of the Board's inquiry into the virus of poliomyelitis, by Dr. Andrewes and Dr. M. H. Gordon.
5. An investigation by Mr. F. W. Twort into the character and life history of certain filter-passing microorganisms.
6. An investigation by Professor Leonard Hill, F.R.S., on respiratory exchange in man under varying conditions.
7. An investigation by Mr. J. E. R. McDonagh on the biochemistry of syphilis.
8. An investigation by Dr. L. Rajchman into the possibilities of serological diagnosis of scarlet fever.
9. An investigation by Dr. D. M. Alexander on the relation between the clinical symptoms and the bacteriology of the acute respiratory affections.

